# Androgenetic Alopecia and Risks of Overall and Aggressive Prostate Cancer: An Updated Systematic Review and Meta-Analysis

**DOI:** 10.3390/cancers17213581

**Published:** 2025-11-06

**Authors:** David G. Hanelin, Sapir Amar, Ilir Agalliu

**Affiliations:** 1 Department of Epidemiology and Population Health, Albert Einstein College of Medicine, Bronx, NY 10461, USA; david.hanelin@einsteinmed.edu (D.G.H.); 2 Yeshiva University, New York, NY 10033, USA; samar1@mail.yu.edu (S.A.); 3 Department of Urology, Montefiore Medical Center, Albert Einstein College of Medicine, Bronx, NY 10461, USA

**Keywords:** androgenetic alopecia, male pattern baldness, vertex balding, frontal balding, prostate cancer, systematic review, meta-analysis

## Abstract

Background: Androgenetic alopecia, also known as male pattern baldness (MPB), is a common hair loss disorder among middle-aged men. MPB shares similar risk factors with prostate cancer (PrCa), including advancing age, family history, and sex hormones. Several studies have examined the associations between MPB and PrCa; however, the evidence remains unclear. We carried out an updated meta-analysis of epidemiological studies that examined the relationship between age at onset and patterns of MPB (either frontal, vertex, or both) and their associations with risks of total and aggressive PrCa. Methods: A literature search was performed using PubMed and Web of Science databases for epidemiological studies published between 1 January 2000 and 31 December 2024 that examined the associations between MPB and PrCa. From each eligible study, relevant data were extracted on study design and population, sample size, prevalence of MPB at various ages, and their association with PrCa. Pooled relative risks (RR) and corresponding 95% confidence intervals (CI) were calculated using the Der-Simonian and Laird random-effects models. Heterogeneity across studies was assessed by I^2^ statistics, while the quality of studies was evaluated using the Newcastle–Ottawa Scale. Results: A total of 19 observational studies, including 17,810 cases and 146,806 controls/non-cases, were analyzed. The prevalence of MPB increased from 5% to 65% with aging and varied across the studies. Both frontal and vertex MPB were associated with a pooled RR of 1.08 (95% CI 1.02–1.14) for total PrCa, but there was no association with frontal-only MPB. Younger-onset MPB (<40 years old) was also associated with an RR = 1.13 (95% CI 0.96–1.31) for PrCa, although results were not statistically significant. Vertex-only MPB was associated with more aggressive PrCa (pooled RR = 1.14; 95% CI 1.02–1.25); however, there was substantial heterogeneity in the definition of aggressive PrCa across the studies. Conclusions: Men with both frontal and vertex MPB have a modestly elevated risk of PrCa. However, most studies were conducted in Caucasian men and they did not evaluate effect modifications by genetic variations in androgen metabolism pathway genes or changes in serum levels of androgens with aging. Large prospective cohort studies with more accurate longitudinal assessment of hair loss patterns are needed to better understand the complex relationship between genetic susceptibility, endogenous hormones, MPB, and subsequent risk of PrCa.

## Simple Summary

Androgenetic alopecia, also known as male pattern baldness (MPB), is a common hair loss disorder in middle-aged and older men, and shares similar risk factors with prostate cancer (PrCa). Several studies have investigated the association between MPB and PrCa, but results have been inconsistent. In this updated meta-analysis of 19 epidemiological studies that includes a total of 17,810 PrCa cases and 146,806 controls/non-cases, we evaluated the relationship between age at onset and patterns of MPB and their association with risks of total and aggressive PrCa. The prevalence of MPB increased from 5% to 65% with aging and varied across the studies. Men with both frontal and vertex MPB had a modest increased risk of PrCa (pooled RR = 1.08; 95% CI 1.02–1.14). Vertex-only MPB was associated with a statistically significant 14% elevated risk of more aggressive cancer. Although men with earlier-onset MPB (aged < 40 years old) had a modestly elevated PrCa risk, results were not statistically significant. Larger prospective cohort studies with accurate longitudinal assessment of hair loss patterns are needed to better understand the complex relationship between genetic susceptibility, MPB, endogenous hormones, and subsequent risk of PrCa.

## 1. Introduction

Prostate cancer (PrCa) is the most frequently diagnosed non-cutaneous cancer in U.S. men and the second most common cancer among men worldwide [1,2]. The well-established risk factors for PrCa include increasing age, race and ethnicity, geographic region, and family history of PrCa [3,4]. Biologically, PrCa is considered androgen-dependent, with both serum dihydrotestosterone (DHT) and testosterone being implicated in the development of this cancer [5,6]. However, pooled meta-analyses of serological studies have reported no associations between endogenous serum levels of either DHT or testosterone and subsequent risk of total PrCa [7,8].

Androgenetic alopecia or male pattern baldness (MPB) affects approximately 30% of Caucasian males by age 30, and its prevalence increases to 50% by age 50 years [9,10]. The development and progression of MPB is mediated by androgens, particularly the potent testosterone metabolite DHT [11]. Androgen potency depends on the receptor binding affinity, with DHT showing approximately five-fold greater affinity than testosterone [12,13]. Prostate cancer and androgenic alopecia share common risk factors, including aging, race/ethnicity, geographic location, and family history/genetics [11,14]. Furthermore, the same sex hormones (e.g., DHT, testosterone) that have been linked to the development of PrCa are implicated in pathogenesis of MPB. Thus, finasteride, a 5α-reductase inhibitor, is widely used in the treatment of androgenetic alopecia to prevent the conversion of testosterone into its more active form, DHT.

Given the above evidence, the question arises as to whether there is an underlying connection between MPB, age at onset, and the development of PrCa. Several epidemiological cohort and case–control studies have investigated the relationship between various patterns of male baldness (e.g., any, frontal, or vertex) with risk of PrCa. Two prior meta-analyses reported that results varied substantially across the studies, with some reporting an increased PrCa risk, while others showing no association [15,16]. The most recent meta-analysis conducted in 2018 [16] reported a statistically significant positive association between vertex MPB and PrCa with a relative risk (RR) of 1.24 (95% CI 1.05–1.46); however, there were no associations for other types of male baldness.

We carried out this updated systematic review and meta-analysis with several goals in mind. We aimed to (1) update the evidence with the most recently published studies since 2018, (2) evaluate further the PrCa risk associated with both age at onset and various patterns of hair loss, (3) identify sources of study heterogeneity and potential for bias, and (4) understand the potential biological mechanisms that interconnect these pathologies and identify gaps for future studies. In this meta-analysis, we included epidemiological studies that were published through 31 December 2024, and investigated risks by both age at onset and patterns of MPB, as well as the associations with total cancer risk and with more aggressive clinical features of PrCa.

## 2. Methods

### 2.1. Search Strategy and Study Selection Criteria

**Figure 1:**
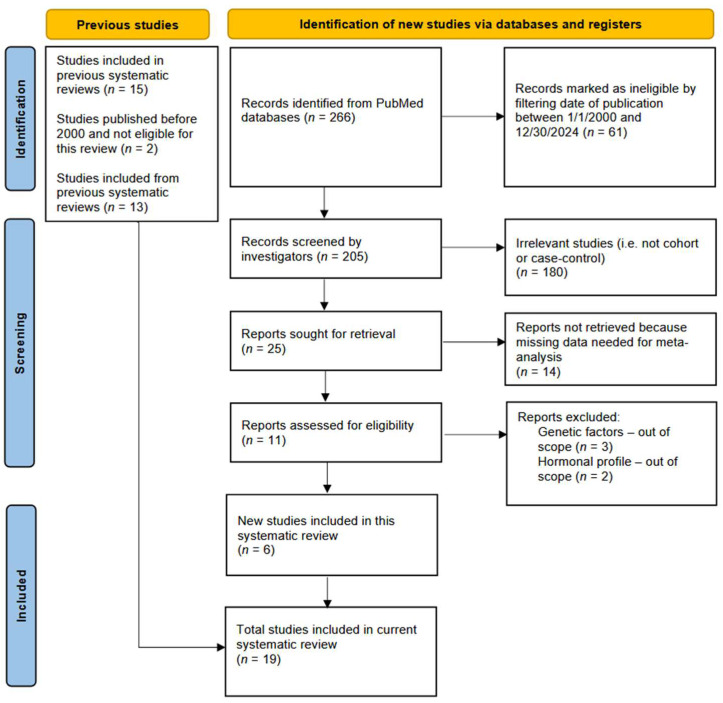
Flowchart of studies selected for the updated systematic review and meta-analysis of male pattern baldness (MPB) and risk of prostate cancer.

A literature search of the PubMed and Cochrane Library databases was performed using the query terms “male pattern baldness OR androgenic alopecia AND prostate cancer”, as well as restricting the publication dates of studies between 1 January 2000 and 31 December 2024. The inclusion criteria were either observational cohort or case–control studies that evaluated the association between MPB or androgenetic alopecia with risk of PrCa. The exclusion criteria were as follows: articles not available in English language, studies involving non-human subjects, studies that were not cohort or case–control design, or those that had missing information on the prevalence of MPB, as well as studies that focused on genetic factors or hormonal profiles associated with androgenetic alopecia, without investigating risk of PrCa. Figure 1 presents the flowchart of studies selected for this updated meta-analysis based on the above inclusion and exclusion criteria.

### 2.2. Data Extraction

All eligible studies that met the inclusion criteria (Figure 1) were carefully evaluated by investigators and all relevant data/information for the systematic review and meta-analyses were extracted (Table 1). This included the first author, year of publication, country where the study was conducted, study design, total sample size (i.e., number of cases and controls or non-cases for each study), patients’ characteristics (e.g., age, race/ethnicity when available), age at MPB onset, the prevalence and patterns of MPB (e.g., frontal, vertex, or both) at various ages, outcomes of interest (PrCa, definition of aggressive cancer), and study results. The MPB was evaluated according to the Hamilton–Norwood classification, which categorizes hair loss patterns in various areas of the forehead and center of the skull (vertex) and ranks hair loss according to the severity of the baldness (see Supplemental Figure S1). Relative risks (RR) or odds ratios (OR) with corresponding 95% confidence intervals (CI) for the association of MPB with PrCa adjusted for potential confounders were also extracted from each study. If studies did not report RR/OR and 95% CI, then we used the data reported in the published manuscripts’ tables to calculate RRs/ORs and their corresponding 95% CI. All data extraction was done according to the PRISMA 2020 guidelines for systematic reviews, which comprises a 27-item checklist [17]. Studies included in our meta-analysis were also evaluated for quality and potential for bias by using the Newcastle–Ottawa Scale (NOS) and other tools for assessing the quality of observational studies [18,19]. The NOS evaluates the studies on three main criteria: selection of participants, comparability between the groups, and ascertainment of exposure (for case–control) or outcome (for cohort studies). The NOS assigns scores for meeting these quality criteria, with a maximum score of nine for the highest quality studies [18]. Studies receiving a score of 7 or higher were deemed of higher quality. The PROSPERO registration number for this meta-analysis is CRD420251053937.

### 2.3. Statistical Data Analyses

We carried out separate meta-analyses and generated forest-plots of studies for the associations of frontal-only, vertex-only, or both frontal and vertex MPB and risks of total and aggressive PrCa using the “metan” command in STATA [20]. The heterogeneity across studies was assessed by the I^2^ statistic, with I^2^ > 50% indicating statistically significant heterogeneity. We used a random-effect model throughout various meta-analyses to account for study heterogeneity. A statistical test with *p* < 0.05 (2-sided) was considered statistically significant. We also carried out stratified analyses by age at MPB onset (<40 vs. 40+ years old) and by study design (cohort vs. case–control studies). The sensitivity analyses were performed by excluding the most influential studies to examine whether the findings in the meta-analysis were robust. Publication bias was investigated by the Begg’s funnel plot trim-and-fill method and the Egger regression test [21,22], with an asymmetric plot suggesting the possibility of publication bias. All statistical analyses were performed by using STATA version 19 (Stata Corporation, College Station, TX, USA).

## 3. Results

A total of 266 records were identified from the PubMed and Cochrane Library databases, of which 241 were deemed ineligible based on screening and filtering criteria, as shown in the PRISMA flowchart Figure 1. Nineteen epidemiological studies [23,24,25,26,27,28,29,30,31,32,33,34,35,36,37,38,39,40,41] published between 1 January 2000 and 31 December 2024 were included in this updated meta-analysis, of which six were newly published articles not included in prior meta-analyses [15,16]. The characteristics of all studies are presented in Table 1, along with a summary of sample size, definition of aggressive PrCa (when available), and the assessment of study quality. Overall, there were seven prospective cohort studies [23,30,34,35,37,38,40] and twelve case–control studies included, with a total sample size of 17,810 PrCa cases and 146,806 controls/non-cases. Most studies were carried out in Europe, the US, and Canada, but three epidemiologic studies were conducted in Australia. On average, men enrolled in these studies were 65 years or older and the majority were Caucasian, with only one study investigating MPB and PrCa among African American men in the US [32]. The definition of aggressive PrCa varied across the studies, with most of them using a Gleason score of 7 to 10 to define more aggressive cancer, but others used either Gleason score 8 to 10, or regional, or distant/metastatic tumor stage, or a serum PSA > 20 ng/mL (Table 1). As anticipated, case–control studies were more likely to be affected by both selection and recall bias and the quality of studies ranged from fair to very good.

**Figure 2:**
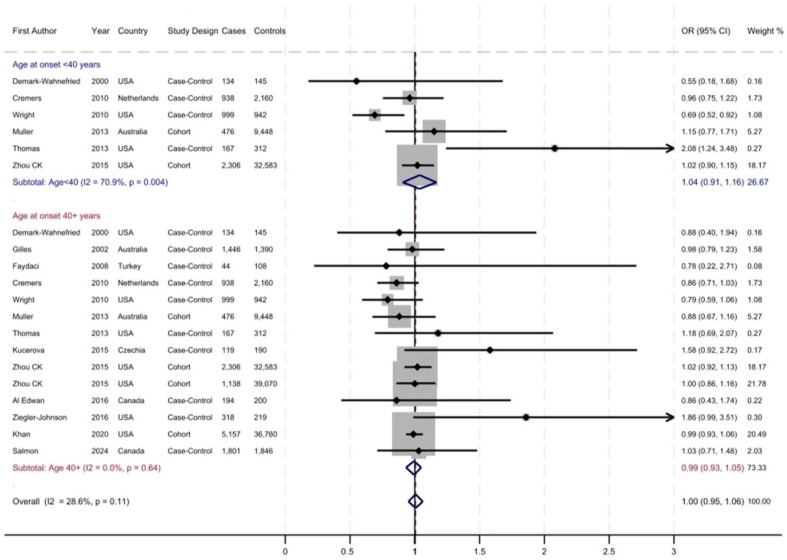
Frontal male pattern baldness (MPB) and risk of prostate cancer. The dotted red line indicates the pooled relative risk (RR).

**Figure 3:**
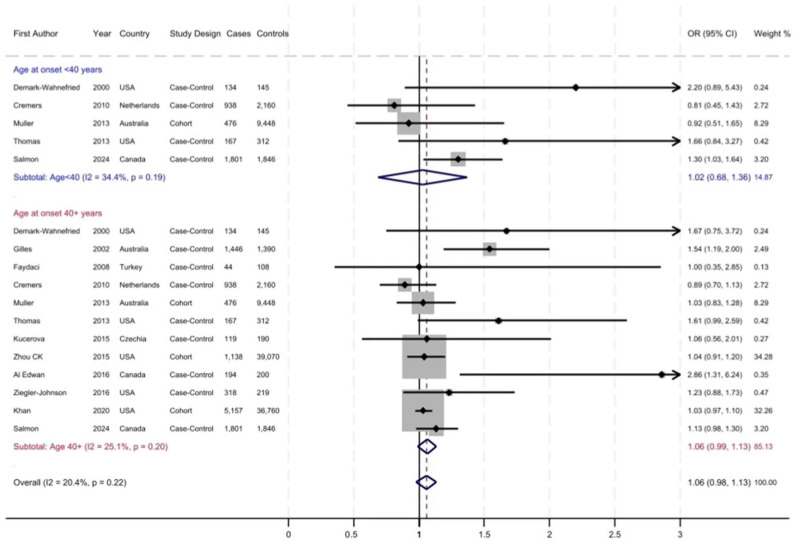
Vertex male pattern baldness (MPB) and risk of prostate cancer. The dotted red line indicates the pooled relative risk (RR).

**Figure 4:**
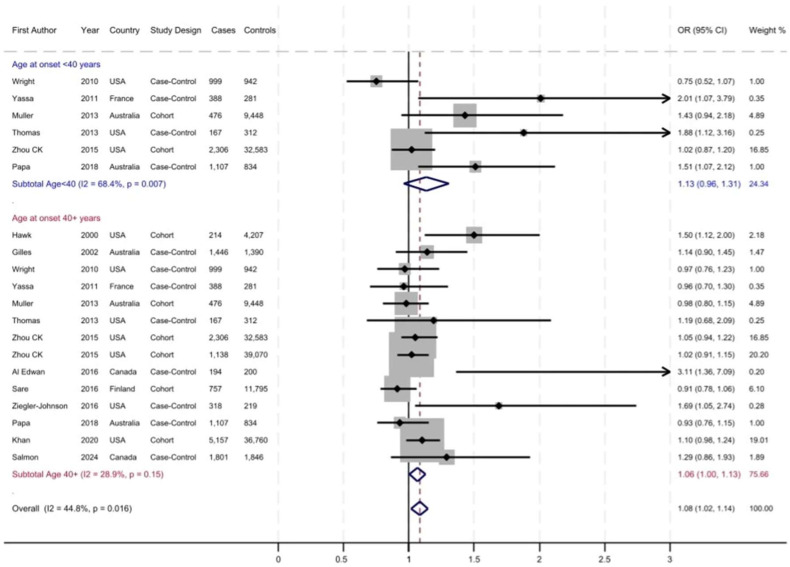
Frontal and vertex male pattern baldness (MPB) and risk of prostate cancer. The dotted red line indicates the pooled relative risk (RR).

The prevalence of MPB increased from 5% to 65% with aging and varied across the studies. Figure 2, Figure 3 and Figure 4 display the forest plots of studies that investigated the associations of patterns of hair loss: frontal-only, vertex-only, or both frontal and vertex MPB, as well as ages at MPB onset with risk of total PrCa, respectively. Overall, there was no association between frontal MPB and PrCa risk (Figure 2 and Table 2). Vertex MPB was associated with a 6% increased risk of total PrCa in men aged 40 years or older (pooled RR = 1.06; 95% CI 0.98–1.13), although results were borderline statistically significant (Figure 3). Among men with vertex pattern hair loss at younger ages there was no elevated risk of PrCa. Both frontal and vertex MPB were associated with a pooled RR of 1.08 (95% CI 1.02–1.14) for total PrCa (Figure 4 and Table 2), but this association was driven mostly by studies with MPB onset at ages 40 years or older. Younger-onset (<40 years) frontal and vertex MPB was also associated with a pooled RR = 1.13 (95% CI 0.96–1.31) of total PrCa, although results were not statistically significant (Figure 4 and Table 2). However, there were only six studies that had available data for this analysis. Notably, there was substantial heterogeneity across the studies with I^2^ values ranging from 20.4% to 70.9% for various meta-analyses, and in some analyses this heterogeneity was statistically significant (Table 2).

We also examined the associations between MPB and aggressive PrCa. Vertex MPB was associated with a 14% higher risk of aggressive PrCa (pooled RR = 1.14; 95% CI 1.02–1.25), but there was no association with frontal MPB (Table 2). However, as shown in Table 1, the definition of aggressive PrCa varied across these studies. Most studies defined more aggressive cancer based on a Gleason score of 7 or higher, while few others also used information on tumor stage, lymph node involvement or metastasis of cancer at diagnosis. A couple of studies also included a diagnostic serum PSA >20 ng/mL or fatal PrCa in this definition. The sensitivity analysis to evaluate differences in results between study designs showed that in general, the association between either vertex-only or both frontal and vertex MPB were stronger and statistically significant in case–control studies (Table 2). By contrast, the associations in cohort studies were weaker, with statistically significant results for the association of both frontal and vertex MPB and risk of PrCa (pooled RR = 1.07; 95% CI 1.00–1.13). Finally, we also evaluated the potential for publication bias. The funnel plot of studies evaluating the association of both frontal and vertex MPB with PrCa risk indicated evidence of this bias with four potential missing studies on the left side of the plot (shown with yellow dots in Supplemental Figure S2). The trim and fill analysis of publication bias showed that if the imputed results of the missing studies were to be added, the pooled RR would be 1.04 (95% CI 1.00–1.09) for the association of both frontal and vertex MPB and PrCa risk.

## 4. Discussion

This updated meta-analysis of 19 epidemiological studies, including six recently published reports, evaluated the evidence of the associations between patterns of hair loss and age at onset of MPB with risks of total and aggressive PrCa among 17,810 PrCa cases and 146,806 controls/non-cases. Our results showed a modest, but statistically significant, positive association between both frontal and vertex MPB and overall risk of PrCa, which was more evident in men reporting onset of MPB at ages 40 years or older. We also found a statistically significant 14% elevated risk of aggressive PrCa associated with vertex-only MPB. However, there was no association between frontal baldness and risk of PrCa. The strength of the association of MPB with PrCa were more pronounced for case–control studies rather than prospective cohort studies indicating that selection and recall bias, as well as potential misclassification, could have affected the results. Notably, there was large statistical variability in results as well as heterogeneity in geographic population and the definition of aggressive PrCa across all studies.

Our analysis builds upon the results of two prior meta-analyses on this topic [15,16]. The first meta-analysis conducted by Amoretti et al. [15] included seven case–control studies and reported a positive association between vertex MPB and PrCa (OR = 1.25, 95% CI 1.09–1.44). In 2018, an updated meta-analysis by He and colleagues [16], which included 15 observational studies (11 case–control and 4 cohort studies), also reported a similar pooled RR of 1.24 (95% CI 1.05–1.46) for vertex MPB and PrCa risk. There were no associations reported for other types of male baldness and PrCa risk. He et al. found positive associations between early age of MPB onset and advanced stage PrCa; however, results were not statistically significant [16]. Similar to our results, the sensitivity analyses of that meta-analysis showed that results were highly influenced by case–control studies [16]. Our updated meta-analysis not only adds to the existing evidence of a link between MPB and PrCa, but also provides a more nuanced and comprehensive understanding of these associations particularly with respect to mixed patterns of MPB. Our age-stratified analysis suggested that late-onset (≥40 years of age) MPB was statistically significantly associated with PrCa risk (pooled RR = 1.06, 95% CI 1.00–1.13). For early-onset hair loss, there was suggestive evidence for an association with PrCa risk, although results were not statistically significant (pooled RR = 1.13, 95% CI 0.96–1.13). Similar results were also reported by He and colleagues for early-onset MPB [16]. To be noted, only 6 out of 19 studies had available data on earlier ages (<40 years) of MPB onset and thus there is limited statistical power for this analysis.

The biological mechanisms linking androgenic alopecia and PrCa likely involve systemic and local androgens metabolism, androgen receptor (AR) signaling, and shared genetic susceptibility. The DHT plays a major role in both MPB and PrCa and functions to promote prostatic cells and tissue growth as well as follicular miniaturization in hair follicles, respectively [5,42,43]. Notably, DHT bioavailability is largely mediated by local intracrine synthesis, rather than systemic circulating levels [44]. In the prostate, epithelial cells can convert adrenal precursors (e.g., DHEA and androstenedione) into DHT, allowing for strong androgenic signaling in the prostate tissue, even as systemic testosterone levels decline with aging. Similarly, at the local follicular level, hair follicles express 5α-reductase enzyme and AR, which allow for local DHT production, resulting in progressive follicular miniaturization [11,12,42,45,46]. This persistent and tissue-specific androgen activity may underline the parallel biology linking these two conditions.

Genetic factors play an important role in this interconnected relationship. Polymorphisms in the AR gene, particularly shorter CAG repeat lengths, have been associated with increased receptor transactivation and linked to both early-onset MPB and higher risk of PrCa [47,48]. Genetic variants in 5α-reductase genes (e.g., SRD5A1 and SRD5A2) may similarly elevate local DHT production, amplifying downstream androgen signaling and enhance local receptor activation [49,50]. Differences in the timing and tissue distribution of androgen exposure may explain the age-related associations in our meta-analysis; e.g. late-onset MPB may reflect prolonged cumulative androgen exposure, whereas early-onset MPB may indicate heightened receptor sensitivity or altered androgen metabolism. Genetic polymorphisms affecting androgen metabolism and/or AR receptor sensitivity/affinity could potentially influence risks of both MPB and PrCa suggesting shared susceptibility [42,45,51]. Although polymorphisms in the AR gene have been linked to early-onset MPB and higher PrCa risk in earlier genetic studies [47,48], recent genome-wide association studies (GWAS) of both MPB and PrCa have identified a large number of genetic variants (SNPs) that modestly contribute to risk suggesting that these pathologies are likely multifactorial and not related to one gene. For example, the GWAS of baldness patterns in Caucasian men have identified many variants that explained 38% of MPB variation, with only one SNP identified in the AR gene [52,53]. By contrast a recent large GWAS of MPB in African men [54] reported that most of the 266 SNPs associated with baldness patterns were autosomal, and the X chromosome did not have a large impact on baldness in African men. Interestingly, none of the studies included in our meta-analysis have investigated genetic susceptibility, or the potential modification that some of the SNPs in the AR or androgen metabolism pathway could have on the risks of MBP and PrCa.

This updated meta-analysis has several strengths, including the large sample size of 17,810 PrCa cases and almost 147,000 controls with the addition of six new studies that were not previously published. We carried out various stratified analyses by MPB pattern, age of onset, and cancer aggressiveness, along with sensitivity analysis by study design and investigation of publication bias. Nevertheless, there are also potential limitations. There was substantial heterogeneity across these studies in terms of design, population characteristics, assessment of MPB, definitions of aggressive PrCa, as well as adjustment for potential confounding. To account for some of this variability, we used random-effects models in our meta-analysis. However, adjustment for confounding was specific to each study (see Table 1), and we used the results provided by the published manuscripts. The definition of aggressive PrCa was inconsistent with most studies relying only on Gleason score, although some studies used both Gleason score, and tumor stage information as well as diagnostic PSA to characterize more aggressive cancer. Additionally, the patterns and degree of hair loss were based only on self-reported information from participants. Although most studies used pictures of hair loss patterns from the Hamilton–Norwood classification, there is still potential for misclassification of specific patterns and age at MPB onset. The possibility for recall bias in case–control studies could also have contributed to both misclassification of MPB and their results. Lastly, most studies included predominantly Caucasian males (Table 1), limiting generalizability to other racially and ethnically diverse groups. The prevalence and patterns of hair loss vary by race and ethnicity [55]. In general, Caucasian men have the highest proportions of androgenetic alopecia with distinct frontal or vertex patterns of hair loss [9,10]. Asian men have the lowest rates of MBP but often present with more diffuse thinning rather than a distinct pattern of receding hairline or crown balding [56]. Finally, men of African descent have higher rates of hair loss, but the pattern is different from Caucasian men [55]. Usually, hair loss in African men begins at the crown and then spread outwards, sometimes preserving the frontal hairline. Some of these nuances might have been lost as most epidemiological studies used self-reported information based on standardized visual pictures of hair loss from the Hamilton-Norwood classification.

## 5. Conclusions

Our updated meta-analysis found that both vertex and mixed-pattern MPB were modestly associated with increased risks of total and aggressive PrCa, particularly in men with MPB-onset at age 40 years or older. There was also a statistically significant association between vertex MPB and aggressive PrCa; however, there was no association between frontal-only baldness and PrCa. Some of the findings could be due to large heterogeneity in results and in the definition of aggressive PrCa across all studies. Moreover, most studies were conducted in Caucasian men, and they did not evaluate effect modifications by genetic variations in the androgen metabolism pathway or other genes, nor changes in serum levels of androgens with aging. Large, well-designed prospective cohort studies with more objective measurement and longitudinal assessment of hair loss patterns and age at MPB onset are needed to better understand the complex relationship between genetic susceptibility, endogenous hormones, MPB, and risk of PrCa. These studies should be carried out in multiethnic populations and should collect data on serum levels of androgens and polygenic risk scores for both MPB and PrCa to better understand the complex relationship between these two pathologies.

## Figures and Tables

**Table 1:** Summary characteristics of the studies included in this updated meta-analysis.

Author	Year	Country	Design	Cases	Controls or Cohort	Age (yrs)	Race/Ethnicity	Definition of Aggressive PrCa	Adjustment Variables for Confounding	NOS Score *
Demark- Wahnefried	2000	USA	Case–control	134	145	60	Cases: W 79%, B 21%; Controls: W 72%, B 28%	NR	Age and race	6
Hawk	2000	USA	Cohort	214	4207	55	W 83%, B 16%, Oth 2%	NR	Age, race, residence, family income (evaluated also education and FH of PrCa)	6
Gilles	2002	Australia	Case–control	1446	1390	84% aged 55 to 69	NR: 31% born outside Australia	GS: 8–10 (high grade)	Age, study center, calendar year, FH of PrCa, and country of birth	6
Faydaci	2008	Turkey	Case–control	44	108	66	NR	NR	None; no logistic model	4
Cremers	2010	Netherlands	Case–control	938	2160	65+ (66% cases), (52% control)	W 98%	Stage ≥ T2c or N+/M+ or GS: 8–10 or PSA > 20	Age and FH of PrCa	7
Wright	2010	USA	Case–control	999	942	60% were 60 to 74	Cases: W 84%, B 16%; Controls: W 90%, B 10%	GS: 7 (4 + 3), 8–10, or regional/metastatic tumor stage, or PSA > 20 ng/mL	Age, race, PSA screening history, FH of PrCa, BMI and finasteride use.	7
Yassa	2011	France	Case–control	388	281	66.5	NR; 16% born outside Australia	GS ≥ 7 or T3/T4 stage	Age and FH of PrCa	5
Muller	2013	Australia	Cohort	476	9448	66	NR	GS > 7 or distant tumor stage	Age and country of birth	7
Thomas	2013	USA	Case–control	167	312	62	W 58%, B 42%	GS ≥ 7	Age, race, FH of PrCa, BMI, and PSA screening history	5
Ziegler-Johnson	2013	USA	Case–control	318	219	59	B 100%	GS ≥ 7	Age	5
Kucerova	2015	Czechia	Cross-Sectional	119	190	63	NR	NR	None	4
Zhou CK	2015	USA	Cohort	2306	32,583	69	7% Other	GS ≥ 7 or regional/distant tumor stage or fatal PrCa	Age, ethnicity, marital status, Charlson comorbidity index, BMI, alcohol, smoking, and aspirin use	7
Zhou CK	2015	USA	Cohort	1138	39,070	71	W 89%, B 3%, Oth: 4%	GS ≥ 7 or regional/distant tumor stage or fatal PrCa	Age, screening arm, study center, education, marital status, diabetes, BMI, smoking, aspirin use, and MI	8
Al Edwan	2016	Canada	Case–control	194	200	63	NR	GS ≥ 7	Age, PSA and DRE abnormalities	4
Sarre	2016	Finland	Cohort	757	11,795	66	NR	GS ≥ 7 or cT3 or fatal PrCa	Age	7
Zhou CK	2016	USA	Cohort	107	4316	54	B 18%	GS: 8–10 (high grade)	Age, race, family income and residence in poverty area	6
Papa	2018	Australia	Case–control	1107	834	65	Majority White	GS ≥ 8 or pT3+ or N1/M1 tumor stage	Age, growth spurt, body shape, ejaculatory frequency, cigarette smoking and alcohol use	6
Khan	2020	USA	Cohort	5157	36,760	60	W 96%, B 1%, Oth 3%	GS: 7 (4 + 3) or 8–10	Age, calendar time, race, height, BMI, FH of PrCa, PSA testing history	8
Salmon	2024	Canada	Case–control	1801	1846	64	W 87%, B 7% Asian 1.3%	GS: 7 (4 + 3) or 8–10	Age, ancestry, education, BMI, smoking status, history of diabetes	7

**Abbreviations**: Race: W—White, B—Black/African American, Oth—other, BMI—body mass index, DRE—digital rectal examination, FH—family history, GS—Gleason score; PSA—prostate specific antigen, PrCa—prostate cancer, NR—Not reported, T-tumor stage (c-clinical, p-pathological), N+/N1—lymph node positive, M+/M1—metastatic cancer. * The Newcastle–Ottawa Scale (NOS) for assessing the quality of observational cohort and case–controls studies in meta-analyses (see methods for details). Studies receiving a score of 7 or higher were deemed of high quality.

**Table 2:** Meta-analyses of male pattern baldness (MPB) and risk of prostate cancer.

Male Pattern Baldness (MPB)	Studies	Pooled RR	95% CI	I^2^ (%)	*p*-het *
**Total PrCa Risk**	Nr				
Frontal-only	15	1.00	0.95–1.06	28.6%	0.11
Vertex-only	12	1.06	0.98–1.13	20.4%	0.22
Frontal and Vertex	14	1.08	1.02–1.14	44.8%	0.016
**Age at MPB onset <** **40 years**					
Frontal-only	6	1.04	0.91–1.16	70.9%	0.004
Vertex-only	5	1.02	0.68–1.36	34.4%	0.19
Frontal and Vertex	6	1.13	0.96–1.31	68.4%	0.007
**Age at MPB onset 40+ years**					
Frontal-only	14	0.99	0.93–1.05	0.0%	0.64
Vertex-only	12	1.06	0.99–1.13	25.1%	0.20
Frontal and Vertex	14	1.06	1.00–1.13	28.9%	0.15
**Aggressive PrCa Risk** **^†^**					
Frontal-only	10	1.03	0.94–1.11	0.0%	0.69
Vertex-only	9	1.14	1.02–1.25	38.8%	0.09
Frontal and Vertex	13	1.07	0.98–1.17	52.6%	0.009
**Case–control Studies**					
Frontal-only	10	0.97	0.86–1.07	39.0%	0.07
Vertex-only	9	1.22	1.07–1.36	39.8%	0.07
Frontal and Vertex	8	1.24	1.07–1.41	70.1%	<0.001
**Cohort Studies**					
Frontal-only	4	1.01	0.95–1.07	0.0%	0.88
Vertex-only	3	1.02	0.93–1.11	0.0%	0.97
Frontal and Vertex	6	1.07	1.00–1.13	41.5%	0.10

* *p*-value for heterogeneity across studies, ^†^ Please refer to Table 1 for the definition of aggressive prostate cancer (PrCa).

## Data Availability

The data used for systematic review and meta-analyses are available upon request from the corresponding author. All relevant studies included in these meta-analyses are already published and data are available through PubMed.

## Supplementary Materials

The following supporting information can be downloaded at: https://www.mdpi.com/article/10.3390/cancers17213581/s1, Figure S1. Hamilton–Norwood classification of male pattern baldness (MPB); Figure S2. Publication bias: funnel plot of studies evaluating the association of both frontal and vertex male pattern baldness (MPB) with overall risk of prostate cancer.
