# Supplementary material for: Androgenetic Alopecia and Risks of Overall and Aggressive Prostate Cancer: An Updated Systematic Review and Meta-Analysis
Source: Cancers (Basel). 2025 Nov 6;17(21):3581. doi: 10.3390/cancers17213581 (PMC12610020; doi:10.3390/cancers17213581)

Supplemental Figure S1. Hamilton-Norwood classification of male pattern baldness (MPB).

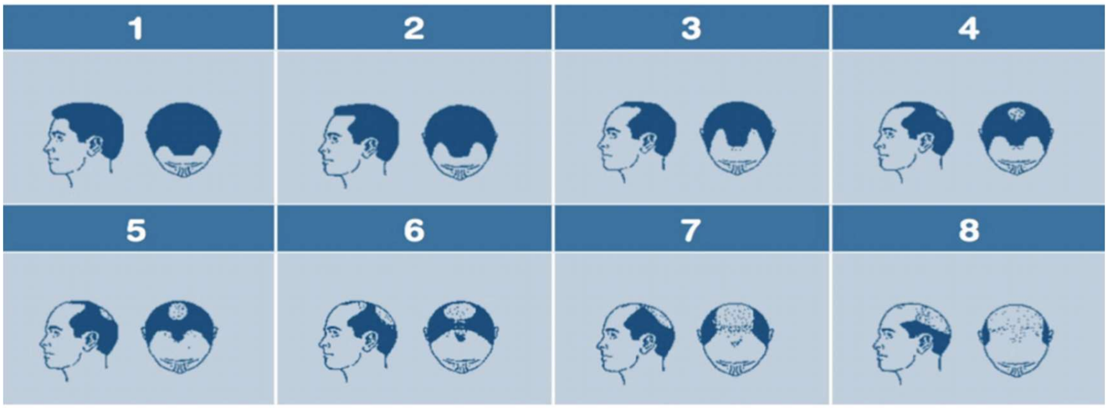

Footnote: In most studies the definition of MPB was done according to the Hamilton-Norwood classification, which categorizes pattern of hair loss in various areas of the forehead and centre of the skull (vertex) and ranks hair loss according to the severity of the hair baldness. Stage 1 indicates no significant baldness, whereas stage 8 indicates severe baldness both frontal and vertex areas.

Supplemental Figure S2. Publication bias: funnel plot of studies evaluating the association of both frontal and vertex male pattern baldness (MPB) with overall risk of prostate cancer

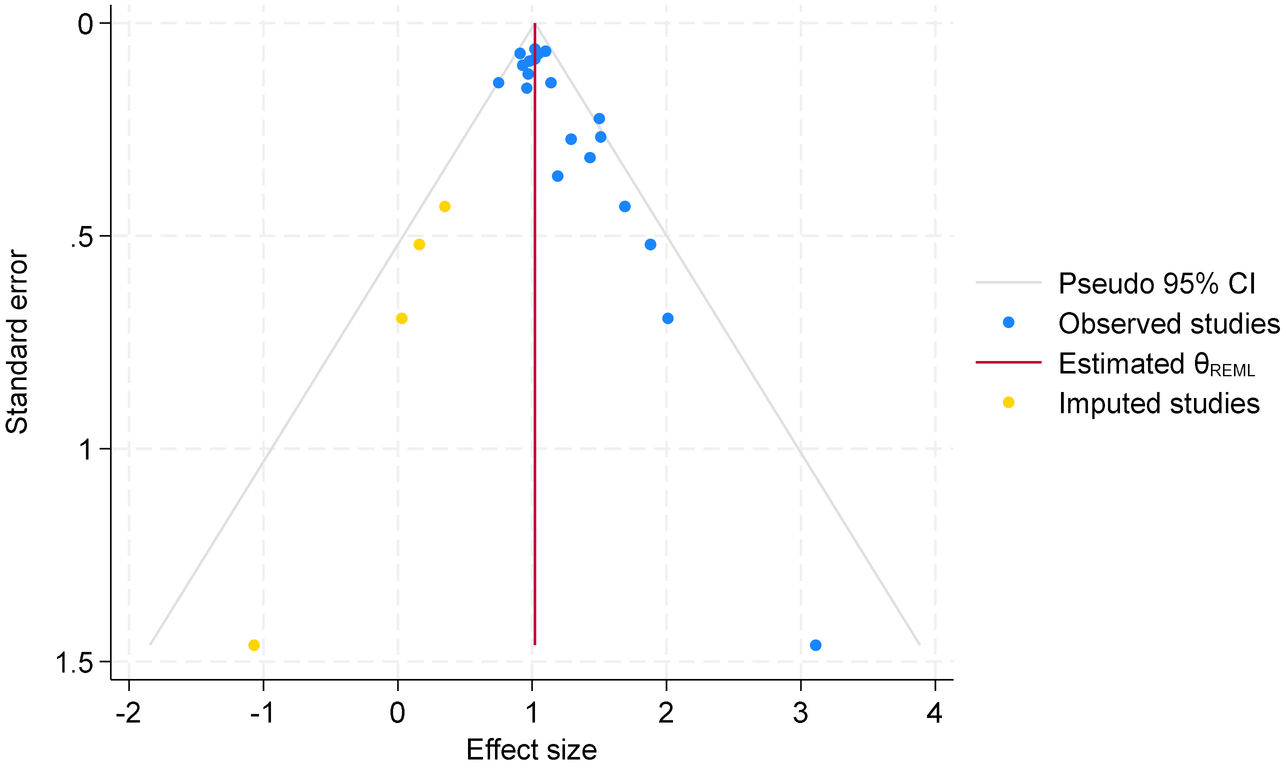

Supplement: Supplementary file 1 [file cancers-17-03581-s001.zip › cancers-3917333-supplementary.pdf]
